# Emergency Presentations to Child and Adolescent Psychiatry: Nonsuicidal Self-Injury and Suicidality

**DOI:** 10.3389/fpsyt.2019.00979

**Published:** 2020-01-17

**Authors:** Monika Franzen, Ferdinand Keller, Rebecca C. Brown, Paul L. Plener

**Affiliations:** ^1^Department of Child and Adolescent Psychiatry and Psychotherapy, University of Ulm, Ulm, Germany; ^2^Department of Ear-Nose-Throat, Vidia Hospital Karslruhe, Karslruhe, Germany; ^3^Department of Child and Adolescent Psychiatry, Medical University Vienna, Vienna, Austria

**Keywords:** nonsuicidal self-injury, self-harm, suicidality, adolescents, emergency, youth

## Abstract

Nonsuicidal Self-Injury (NSSI) and suicidality are common reasons for emergency presentations in child and adolescent psychiatry. Therefore, we focused on reasons for emergency presentations as well as specific characteristics of those presenting with NSSI or suicidality to an emergeny psychiatric service. We analyzed data from a German university hospital regarding emergency presentations during a 78 months' period. NSSI and suicidality were rated according to the Columbia Classification Algorithm of Suicide Assessment (C-CASA). Data from 546 emergency presentations was recorded, of which 347 (63.5%) presented for NSSI or suicidality. Given the high percentage, thorough assessment of sucidality as well as providing adequate treatment in emergency settings to establish further care, is of utmost importance.

## Introduction

Nonsuicidal Self-Injury (NSSI), defined as repetitive direct self-inflicted damage of one's own body surface without suicidal intent (1), has been described repeatedly as common phenomenon in adolescents, with rates ranging between a lifetime prevalence of 18%–28% in international school samples (2, 3). In clincial samples, rates of NSSI have been reported to be even higher, reaching up to 50% in child and adolescent psychiatric inpatients (4). With regards to suicidality (including both suicidal ideation as well as suicide attempts), rates of suicidal ideation have been reported to be between 19.8% and 24% in youth (5), whereas lifetime prevalence rates between 3.1% and 8.8% have been reported for suicide attempts in minors (5). Germany is—within Europe—among the countries with the highest rates for NSSI in adolescent scool samples, with a lifetime prevalence of 35.1% (3). In addition, a lifetime prevalence rate for suicidal ideation of 39.4% has been reported from a representative sample of German high-school students, with 9% reporting a lifetime suicide attempt (6).

The German clincial guidelines for NSSI and for suicidality in youth recommend assessments for acute suiciality by a child and adolescent psychotherapist or a specialist for child and adolescent psychiatry both in patients presenting with NSSI, as well as in patients with suicidality (7, 8). Therefore, children and adolescents, who report NSSI and suicidality often present themselves to child and adolescents psychiatric emergency services as a first contact to the mental health system. Despite the high prevalence rates and the high clincial relevance of these presentations, so far, only a limited amount of scientic literature has been published about the prevalence and nature of these presentations in child and adolescent psychiatric settings.

An analysis of 1,093 emergency presentations to an Austrian child and adolescent psychiatric service showed suicide attempts to be the most prominent reason (22%) for emergency contacts in female minors with a migration background and the second most common reason in females without migration background (9). A crucial number of similar presentations was also reported from an analysis of 328 mental heath emergencies (in 179 patients) presenting to a pediatric department in Spain (10). Self-harm behavior (defined as intentional act of self-poisoning or self-injury irrespective of degree of suicidal intent) was the second most common reason for presentation in females (29%), following behavior disorder (30%) in rank. In an analysis of clinical data from a Canadian service provider, 468 cases of minors presenting with mental health emergencies were analysed. NSSI within the previous 24 h was recorded in 45% of all cases. Of these, 91% were NSSI only, whereas 5% reported a suicide attempt and 4% involved co-occurring NSSI and suicidality. Those with co-occuring NSSI and suicide attempts showed the highest level of psychopathology (11). In an analysis of 181 youth presenting to an emergency department with suicidal ideation or a suicide attempt, a high overlap between judgement about level of sucidality from the parent´s and the patient´s point of view was described (12)

Given the paucicity of data in this clinically relevant field of child and adolescent psychiatric emergency service provision, we aimed to systematically assess acute child and adolescent psychiatric presentations based on a retrospective chart review. Focusing more specifically on NSSI and suicidality, we decided to classify these incidents based on the Columbia Classification Algorithm (C-CASA). Since emergency presentations call for timely reactions providing a high level of safety, it is of importance to better understand dynamics of these presentations. These analyses could inform a tailored planning of interventions with regards to availability of staff, the need for pediatric health care (e.g., in intoxications) or provision of risk assessment. Given the importance of school in adolescents' daily life, we hypothesized that time patterns of presentations will be influenced by the school year (with lower presentations during the summer and higher number of presentations during exam times). Thereby, we aimed at answering the following questions:

- What are the reasons for which children and adolescents present to child and adolescent psychiatric emergency services?
- What are the specific characteristics of presentations for suicidal behavior or NSSI?

## Materials and Methods

We conducted a retrospective chart review of all children and adolescents presenting to the emergency services at a German University Department of Child and Adolescent Psychiatry and Psychotherapy. The Department serves a region of 402,000 inhabitants as sole provider of inpatient care and emergency child and adolescent psychiatry 24 h for 7 days a week. Electronic records were searched for presentations between 5 pm and 8 am as well as on weekends (outside office hours). Our inclusion criteria were: 1) children and adolescents below the age of 19; 2) Presentation as emergency contact outside of regular working hours to the Department of Child and Adolescent Psychiatry and Psychotherapy at the University of Ulm; and 3) Date of presentation between 01.06.2006 and 31.12.2012.

Based on a search of the hospital´s electronic database, we retrieved each patient record from all patients, who were fulfilling the abovementioned inclusion criteria and aimed to sort information following a pre-defined schedule of search items. Based on the information stored in the records, we classified the cause for the presentations at an emergency child and psychiatric service. In case of presentations, in which self-injuring or sucidal behavior was listed in the context of the emergency presentation, we applied the Columbia Classification Algorithm of Suicide Assessment (C-CASA) classification algorithm. This algorithm was originally developed to classify suicidal behavior in medication trials in minors (13). Given the retrospective nature of the application of this algorithm to pre-existing records, we found it to be perfectly fitting to our approach of data collection and classification. The C-CASA allows for classification in several categories:

- Suicidal (completed suicide, suicide attempt, preparatory action, suicidal ideation)
- Nonsuicidal (nonsuicidal self-injury, other: accidental, psychiatric, medical)
- Indeterminate (self-injury with unknown intent, not enough information)

The original study showed an excellent interrater reliabilty (mean ICC: 0.89) between nine raters (13). In our study, data about those presenting as emergency cases was retrieved and caseness was defined by a consultant child and adolescent psychiatrist. C-CASA classification was first provided by a resident MD, trained with general C-CASA cases. Cases were then presented to a consultant child and adolescent psychiatrist for review. This retrospective chart review was in accordance with the Declaration of Helsinki and was approved by the IRB of the University of Ulm.

## Results

Within the searched 78 months' period N = 546 cases (mean age: 14.43; SD: 2.43; age range: 10–17; female: 56.2%) fulfilled the aforementioned criteria and were included in our analysis. Emergency contacts were not evenly distributed across the weekdays (χ^2^ = 18.04; p = 0.006), but tests of each weekday against deviation from the expected value of 78 (14.3%) contacts per weekday revealed that only Saturday (51 = 9.34%) was significantly different (χ^2^ = 9.50; p = 0.001). Concerning the month of presentation, there is no overall difference across the months (χ^2^ = 16.07; p = 0.139). In single tests, however, there are tendencies for June (58 = 10.6%) having a higher number (χ^2^ = 3.37; p = 0.067) and August (33 = 6.0%) having a lower number (χ^2^ = 3.57; p = 0.059) than the expected number of presentations of 45.5 (8.3%) per month. Of these contacts, 33.7% were discharged at the same or the following day. The mean treatment duration of those, who were admitted to inpatient care was 28 days (median: 5 days).

With regards to the primary diagnoses, most patients (36.6%) received a diagnosis from the ICD-10 diagnostic group F90–F98 (Behavioural and emotional disorders with onset usually occurring in childhood and adolescence). This was followed by diagnostic group F30–F39 (mood disorders) with 25.5% and F40–F48 (Neurotic, stress-related and somatoform disorders) with 15.3%. While only a minority of patients were receiving psychopharmacological treatment at the time of their emergency contact (12.45%), 43% received a prescription after their contact, with antipsychotics (21.6%), antidepressants (16.2%), and stimulants (8.4%) being the most widely prescribed psychopharmacological agents, with more than one agent prescribed in 102 patients.

We further analysed those patients, who fulfilled at least one category of the C-CASA scheme (n = 347, 63.5% of the whole sample) as reason for presenting to child and adolescent psychiatric services (see **Table 1**). Overall, in each C-CASA category, more female than male patients presented themselves as emergency contacts, with significant sex differences in suicide attempts (χ^2^ = 9.94; p < 0.001), preparatory acts (χ^2^ = 4.41; p = 0.014), suicidal ideation (χ^2^ = 22.02; p < 0.001), and NSSI (χ^2^ = 49.88; p < 0.001).

**Table 1:** C-CASA classification (n = 347).

Category	Number (%)	Female sex (%)
C-CASA 1: Suicidal		
Suicide	0	
Suicide attempt	49 (14.12)	38 (77.55)
Preparatory acts	45 (12.97)	32 (71.10)
Suicidal ideation	120 (34.58)	90 (75.0)
C-CASA 2: Nonsuicidal		
NSSI	127 (36.6)	106 (83.46)
Other nonsuicidal self-harm	2 (0.58)	2 (100.0)
C-CASA 3: Undetermined		
Self-injury undetermined intent	4 (1.15)	3 (75.0)
Not enough information	0	

NSSI led to 127 presentations, therefore accounting for 22.52% of all emergency contacts, with youth being predominantely female. Age curves showed two peaks at age 14 and age 16 for presentations.

Presentations for sucide attempts were highest on Mondays (24.49%) and in October (14.28%). Controlling for holidays, we found more presentations with suicide attempts outside of national holidays (p = 0.042). The methods recorded for suicide attempts included (in order of decreasing likelihood): intentional self-poisoning with non-psychopharmacologcial agents (X60+X63) with 32.2%, self posisoning with other substances (X64) with 15.25%, and self-poisoning with psychopharmacological and hallucinogenic agents (X61+X62) with 11.86%. Intentional self-harm with a sharp object (X78) was reported in 15.25%, whereas 8.47% reported intentional self-hanging (X70).

## Discussion

We conducted a retrospective chart review of children and adolescents presenting to a child and adolescent psychiatric emergency service. Our analyses included reasons for presentations as well as further characteristics of the patients and the nature of presentation. We were able to show that NSSI and suicidality are the main reasons for presenting to our child and adolescent psychiatric services. This seems in line with former studies on child and adolescent psychiatric services or pediatric services providing emergency mental health care (9, 10, 14), which also underlined the clinical relevance of these topics for emergency child and adolescent psychiatry. Looking into methods used for suicide attempts, self-poisoning, as well as self-harm with a sharp object and self-hanging was reported. Intoxications are among the most frequently reported methods for suicide attempts in adolesents (5), and intentional self-harm is a common and rising phenomenon in adolescents, also in community samples (15). Self-hanging is the most common method of suicide in the age groups 10–19, whereas there are hardly any suicides in this age groups commited by guns (X72–X74) (16). Although presentations for suicide attempts, represent the three most prevalent methods for suicide attempts in youth (5), it has to be noted that the absence of presentations involving firearms is likely due to legal restrictions on German gun law and may not be comparable to the situation in other countries. We were unable to describe temporal monthly patterns for emergency presentations, although a tendency for more admissions in June and less admissions in August was observed. This pattern could point to an influence of schooling on emergency psychiatric contacts, as June is at the end of the German school year, thus often leading to high work-loads and exam pressure, whereas in August schools are closed for holidays. Interestingly, fewer emergency contacts were observed for Saturdays, which is also a school-free day in Germany.

Given that NSSI and suicidality were found to be among the main reasons for presenting at a child and adolescent emergency psychiatric service, it seems crucial to provide efficiant services at that point of care. This includes thorough assessment (17), as well as adequate follow-up care. Especially in the population of minors with NSSI, high barriers to seeking treatments have been reported (18, 19). It seems crucial to lower these barriers to make healthcare accesible to those with urgent needs, that are in a possibly life-threatening state. This could be achieved by presenting information about child and adolescent psychiatric services in youth-friendly way (such as for example brochures^1^), by anti-stigma work or by increasing awareness of mental health problems and care providers as well as by increasing mental health literacy (20). Furthermore, adolescents could profit from online interventions and services to lower barriers to treatment. It has been discussed, that the admission to emergency care due to suicidal behavior can be a stressful situation for those seeking acute care (21), especially if specialized services for youth are lacking (22). Youth stressed the importance of information and compassionate clinicians in this situation, and stated that repeated questioning from different clinicians was perceived as negative (23). Due to the retrospective nature of our analysis, we were not able to provide information about patients´ experiences with emergency psychiatric care, although this would clearly help to inform future optimization of assessment and treatment in these situations from a customer´s viepoint. Given the high number of patients, who access emergency psychiatric care, it is of importance to standardize assessment and procedures, allowing for high levels of safety, reduce waiting time and create a flow of information between outpatient assessment and inpatient treatment in those situations where inpatient admissions are necessary.

In addition, it is of utmost importance to make the first contact of young patients to service providers in an acute situation as engaging as possible to secure follow-up care, also in situations in which follow-up is provided in an outpatient setting. This could be achieved by implementing strategies such as Therapeutic Assessment (24), which was designed to engage children and adolescents presenting with NSSI and suicidal behavior in follow-up mental health care. It has been shown, that the likelihood of presenting to follow-up care and the duration of participating in follow-up care can be increased by a simple and short intervention at the first contact (24). Given that there is an increasing evidence for the efficacy of psychotherapy to decrease NSSI or suicidal behavor (25) in youth, efforts must be undertaken to use the first contact to mental health care providers to engage minors in further care.

Following up on this retrospective analysis, future research should focus on the adolescents´ and caregivers´ experience of emergency presentations to child and adolescent psychiatry and use these informations to create optimized procedures, which could result in lowering the threshold of seeking specialist care in mental health crisis situations. Although data about whether adolescents sought help themselves, or were brought to the psychiatric department by their caregivers is not available from our dataset, this could influence willingness to participate in further mental health care and should therefore be assessed in future studies. Building on this information, specific procedures could be tested for their outcome in patient and caregiver satisfaction, as well as for their outcome concering patient safety and further engagement in mental health follow-up care.

## Limitations

We presented data of one department situated in Germany, therefore limiting generalizability of our findings for other settings or countries. We defined emergency presentations as occuring outside of office hours. This neglects the fact that emergency presentations are also possible during office hours. This restriction was based on a lack of possibility to retrieve information about the nature of presentation (acute vs. planned) from retrospective records. We therefore chose to limit our analysis to an intake time, which restricts presentations to those with an acute nature. This approach was based on literature showing that NSSI most often is happening throughout evening hours (26). We are aware that our analysis therefore presents a rather conservative evaluation of emergency contacts, which has to be taken into account when interpreting these findings. Due to the retrospective nature of the study, standardized risk-assessment was not available, creating a risk for misclassification. Therefore, future analyses could benefit from a standardized mean of risk assessment at intake.

## Conclusions

NSSI and suicidal behavior account for a majority of emergency presentations in child and adolescent psychiatry. Therefore, there is a need to put a special emphasis on these situations in the clinical training of residents, as well as provide the best care possible in this specific situation. Apart from providing a thorough risk exam, this should also include motivational aspects, such as in Therapeutic Assessment, among other possible approaches, to increase the likelihood of minors to participate in follow-up care.

## Data Availability Statement

The datasets generated for this study are available on request to the corresponding author.

## Ethics Statement

The studies involving human participants were reviewed and approved by the ethical committee of the University of Ulm. Written informed consent from the participants' legal guardian/next of kin was not required to participate in this study in accordance with the national legislation and the institutional requirements.

## Author Contributions

MF conducted the retrospective chart review, with help from FK and PP. PP and RB drafted the manuscript. All authors read and approved the final manuscript.

## Funding

Data were collected as part of a study funded by the Department of Child and Adolescent Psychiatry at the University of Ulm.

## Conflict of Interest

PP has received research funding from the German Federal Ministry of Research and Education (BMBF), the German Federal Agency for Drugs and Medical Products (BfArM), the Baden-Wuerttemberg Foundation, Lundbeck, Servier and the Volkswagen Foundation. RB has received funding from the Baden-Wuerttemberg Foundation.

The remaining authors declare that the research was conducted in the absence of any commercial or financial relationships that could be construed as a potential conflict of interest.

## Footnotes

1 https://www.uniklinik-ulm.de/fileadmin/default/Kliniken/Kinder-Jugendpsychiatrie/Dokumente/WebversionWIDSN.pdf (accessed on September 24th, 2019).
